# Supplementary material for: Simultaneous UHPLC-MS Quantification of Catechins and Untargeted Metabolomic Profiling for Proof-of-Concept Authenticity Determination of Maytenus ssp. Samples
Source: Molecules. 2022 Aug 27;27(17):5520. doi: 10.3390/molecules27175520 (PMC9457526; doi:10.3390/molecules27175520)
Supplement: Supplementary file 1 [file molecules-27-05520-s001.zip › molecules-1858367-supplementary.pdf]

Supplementary

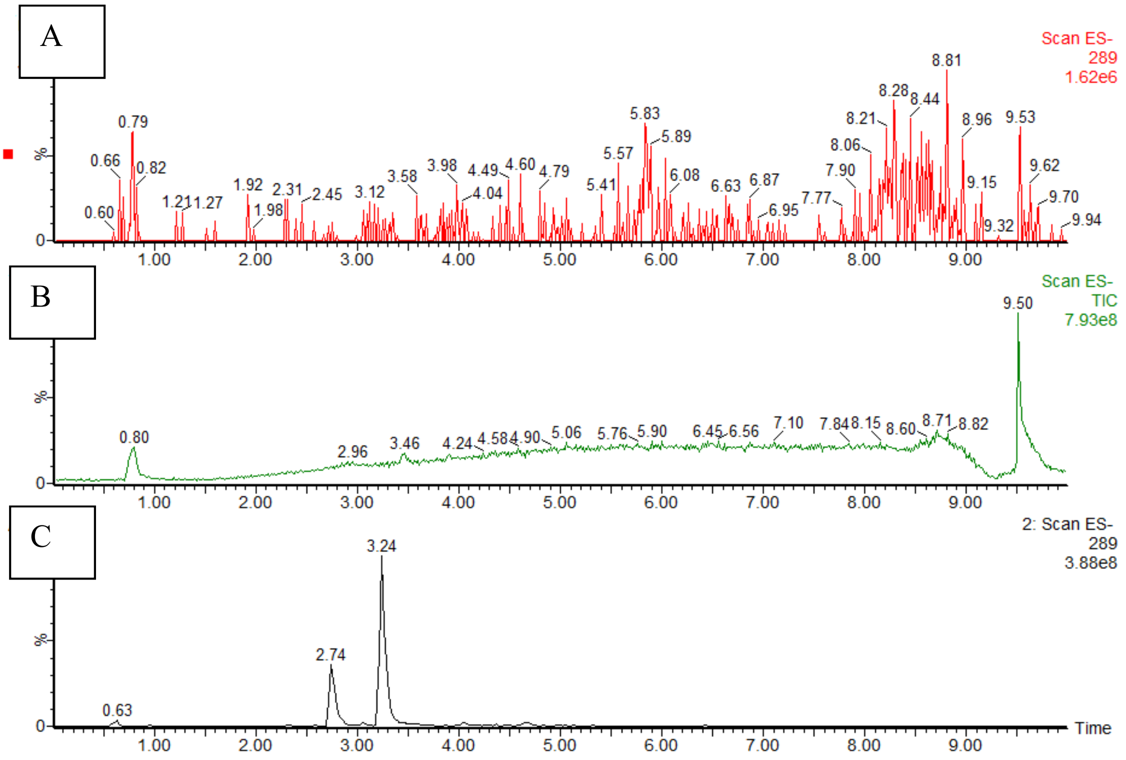

Figure S1. UHPLC-MS chromatograms acquired in A- SIM of the solvent, B- full scan of solvent and C- SIM of the standards.

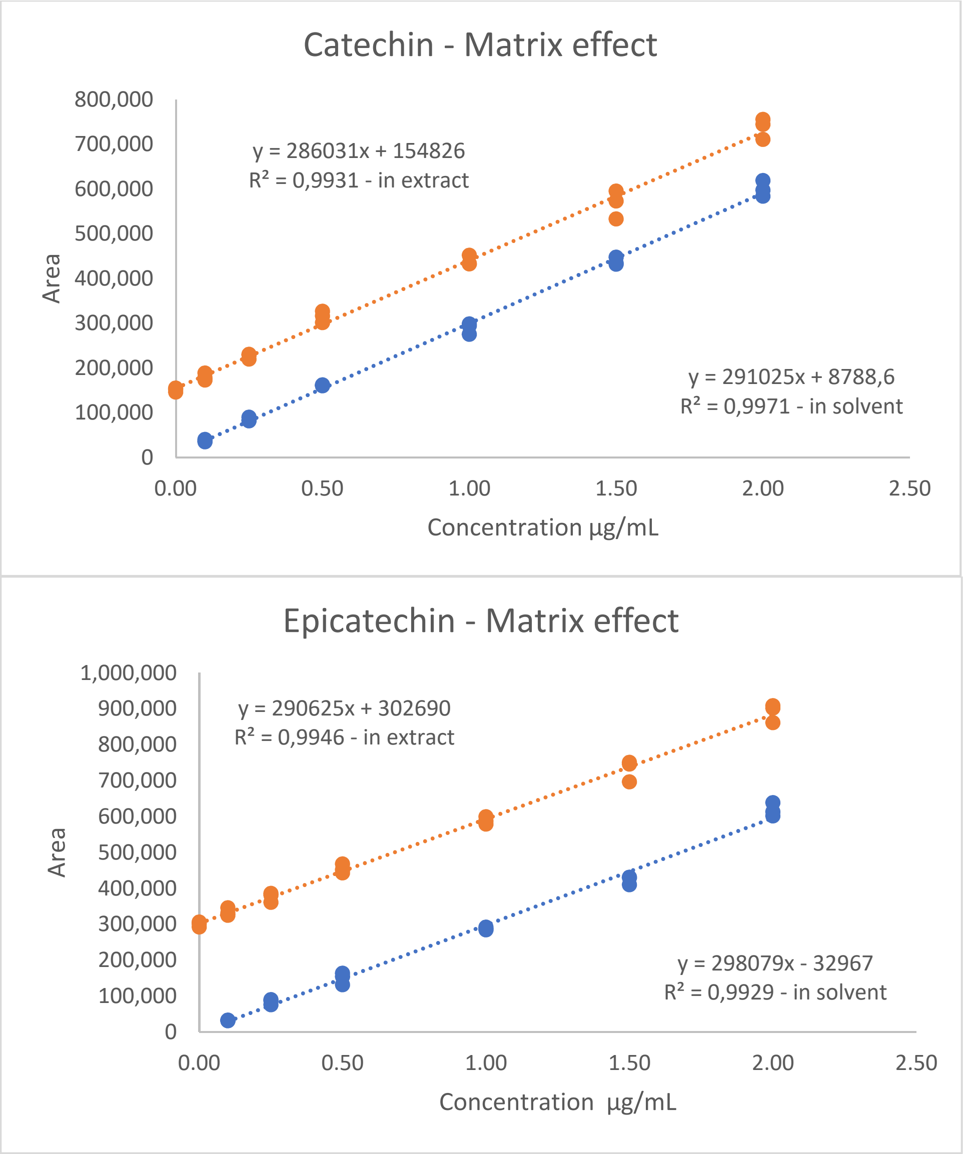

Figure S2. Compared analytical curves of catechin and epicatechin in extracts and solvent.

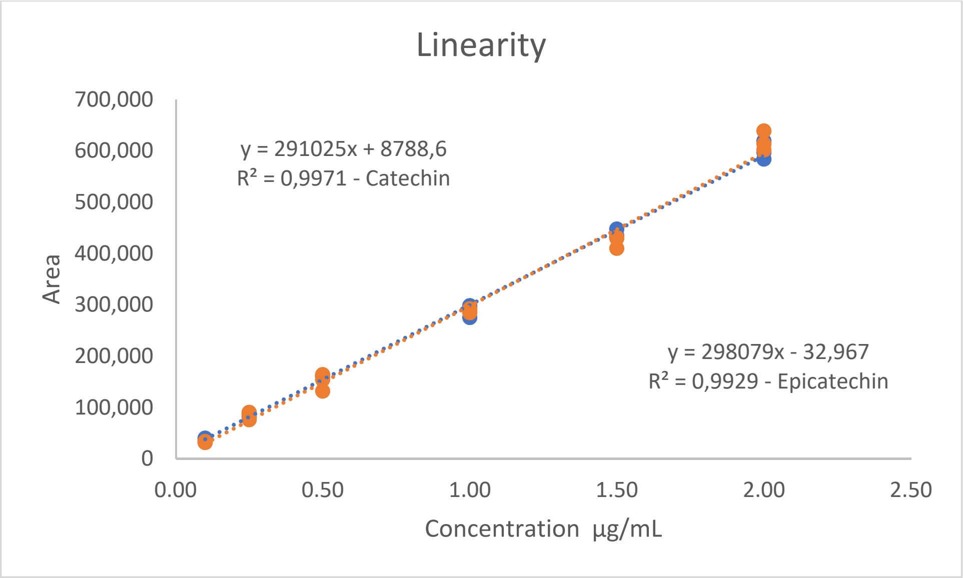

Figure S3. Analytical curves of catechin and epicatechin

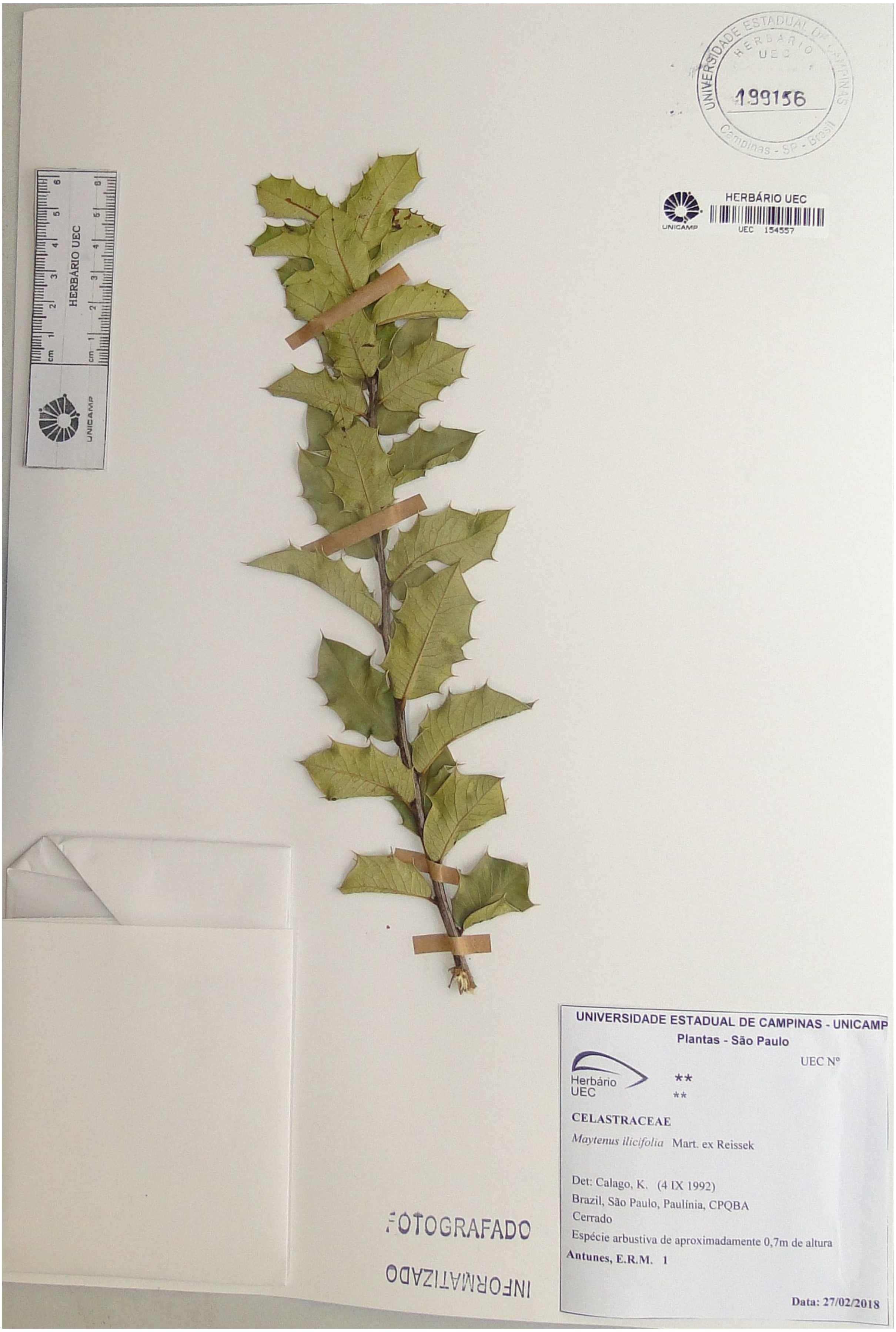

Figure S4. Voucher of the authentic *Maytenus ilicifolia* sample (UEC 199156)

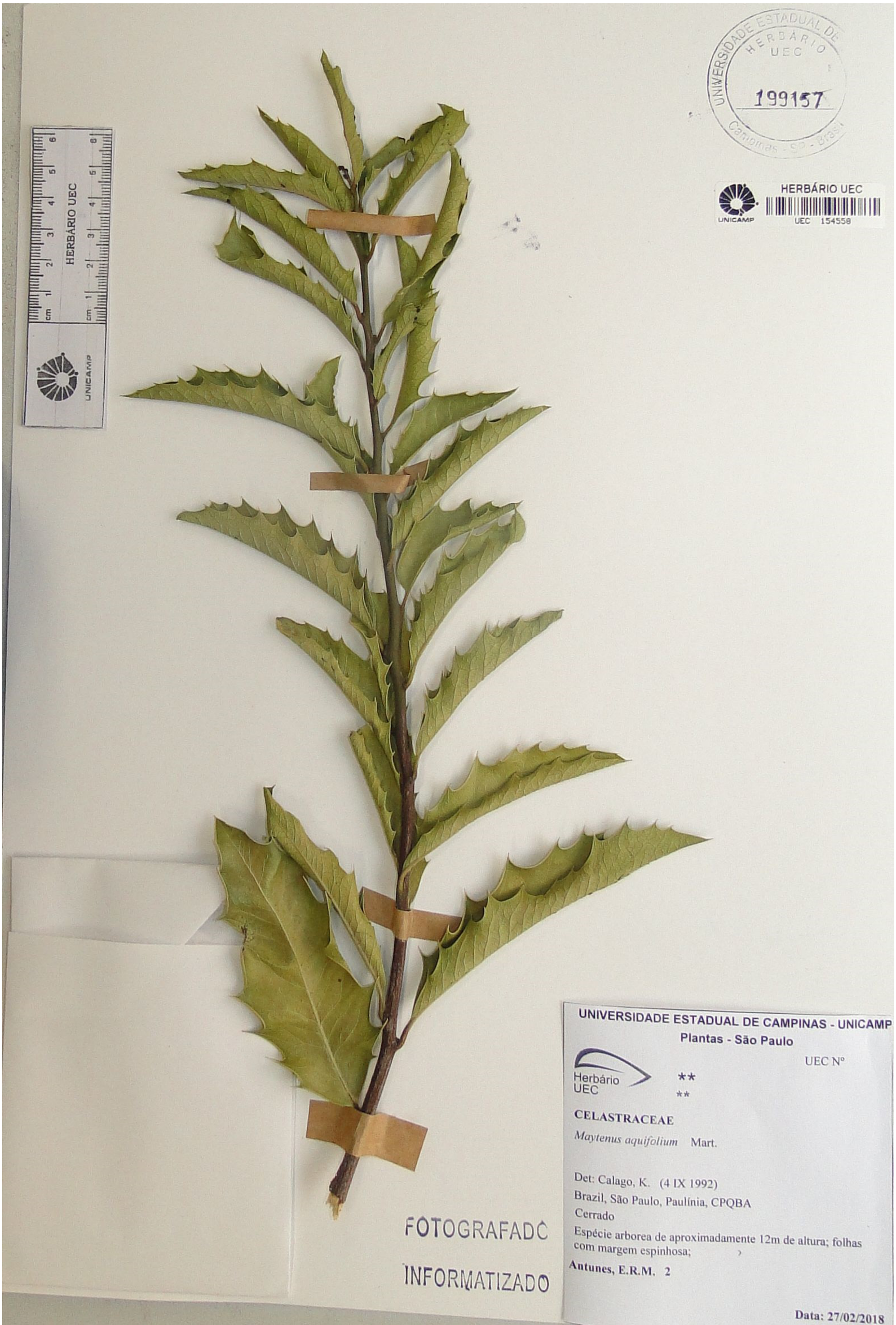

Figure S5. Voucher of the authentic *Maytenus aquifolium* sample (UEC 199157)

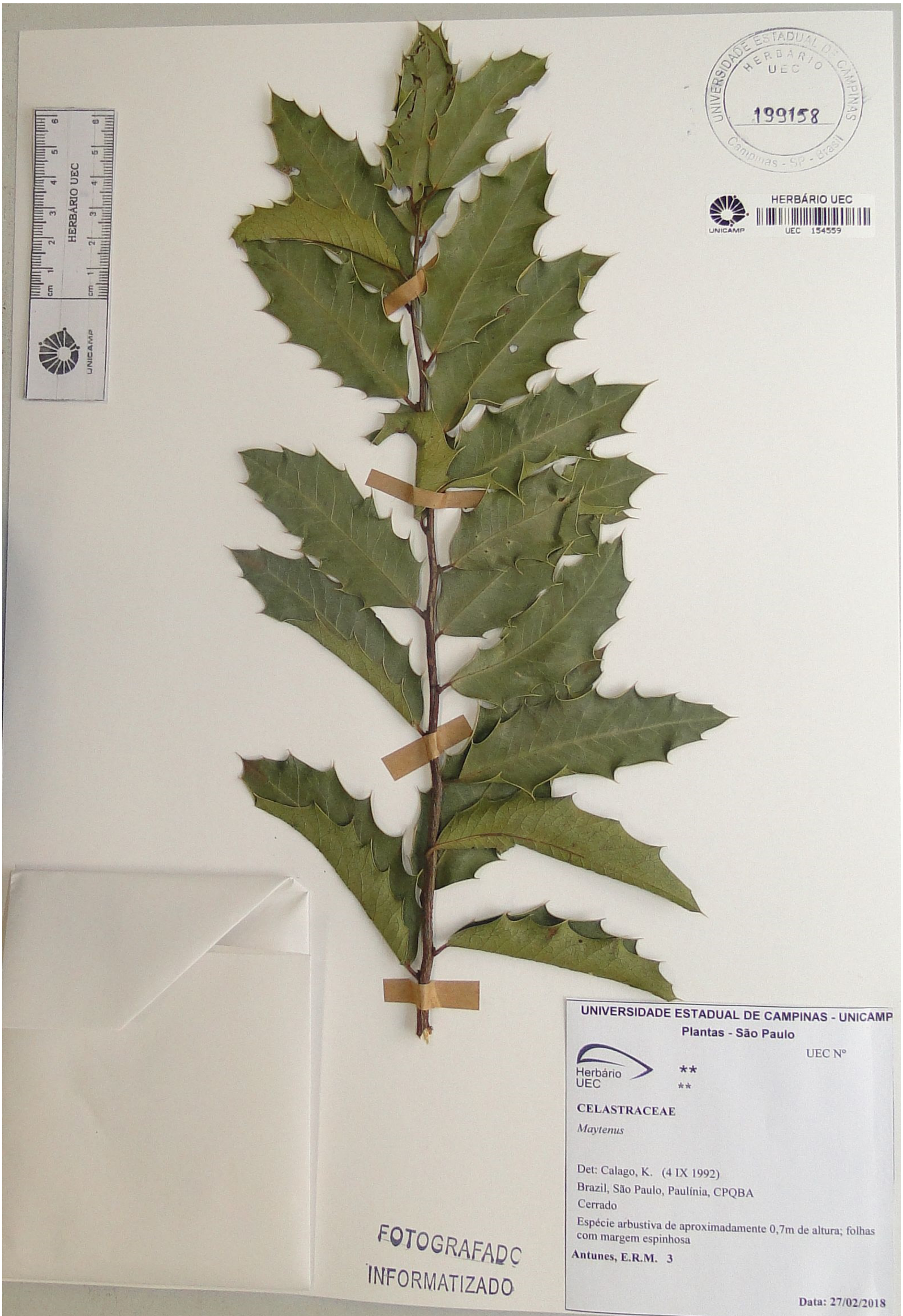

Figure S6. Voucher of the hybrid *Maytenus* sample (UEC 199158)

Table S1. Repeatability and intermediate precision of the method using calibration curves: catechin (y = 289268x + 12722) and epicatechin (y = 294056x + 10672).

REP	Catechin [μg/mL]	Epicatechin [μg/mL]	Catequina [μg/mL]	Epicatequina [μg/mL]
	Repeatability		Intermediate precision	
1	4.22	8.94	4.31	9.05
2	4.62	8.38	4.96	9.83
3	4.30	8.67	4.10	8.75
4	4.36	9.33	4.31	9.64
5	3.78	9.25	4.65	8.84
6	4.31	8.90	4.31	8.66
Average	4.26	8.91	4.44	9.13
SD(σ)	0.28	0.36	0.31	0.49
RSD %	6.5	4.0	7.0	5.4

Table S2. Recovery of Catechin in three levels

**Theoretical concentration (μg/mL)**	**Measured concentration (μg/mL)**	**RSD %**	**Recovery %**
0.1	0.11	13.89	109.33
1.0	0.98	3.84	98.50
2.0	2.00	3.98	100.12

Table S3. Recovery of Epicatechin in three levels

**Theoretical concentration (μg/mL)**	**Measured concentration (μg/mL)**	**RSD %**	**Recovery %**
0.1	0.10	14012	103.12
1.0	0.97	3.50	97.90
2.0	2.03	2.06	101.40

Table S4. Detection limit (DL) and quantification limit (QL) of Catechin and Epicatechin

	**Linear equation**	**DL (μg/mL)**	**QL (μg/mL)**
Catechin	Y=289428x + 4732.4<br>Y=288962x + 3771.8<br>Y=283573x + 6011.1	0.03	0.1
Epicatechin	Y=303439x + 3808.7<br>Y=298011x – 1998.4<br>Y=297270x + 4165.5	0.03	0.1

Table S5. Extraction and analytical conditions which were varied and recovery of Catechin and Epicatechin (%)

**Condition**	**Catechin (%)**	**Epicatechin (%)**
Centrifuged	100.0	100.0
PVDF 0.22 μm filter	97.8	103.4
Nominal/ validated	100.0	100.0
Flow 0.19 mL/min	97.8	95.7
Flow 0.21 mL/min	100.7	97.4
Temperature 25°C	100.9	97.1
Temperature 35°C	97.7	98.0
Solv. A 0.5% formic acid	95.5	95.1
Solv. A 1.5% formic acid	101.2	96.7
A different C18 column	99.9	96.9
